# The Impact of Experience on Motion Information Processing: An ERP Study

**DOI:** 10.3390/bs16020284

**Published:** 2026-02-15

**Authors:** Yinan Xu, Xue Sui

**Affiliations:** 1 The School of Physical Education, Liaoning Normal University, Dalian 116029, China; xuyinan0412@163.com; 2 The School of Psychology, Liaoning Normal University, Dalian 116029, China

**Keywords:** experience, sports information, event-related potential (ERP)

## Abstract

The purpose is to investigate how sports experience influences the processing of motor-related information. Sixty participants with differing levels of sports experience were recruited: 20 table tennis athletes, 20 athletes from other sports, and 20 non-athletes. A total of 150 images depicting table-tennis scenarios, divided into competitive and non-competitive, were shown to participates and recorded their electroencephalographic responses. We found that both table tennis and ordinary athletes exhibited significantly smaller P3 amplitudes in the parietal region compared with non-athletes. In addition, under competitive conditions, athletes showed larger N2 amplitudes in the central region than non-athletes. However, no significant difference in N2 amplitude was observed between table tennis athletes and athletes from other sports. These findings indicate that greater sports experience reduces the cognitive resources required for processing motor-related information and enhances individuals’ abilities in conflict monitoring and response inhibition. Furthermore, the effects of sports experience appear to be transferable across different athletic domains.

## 1. Introduction

In everyday life, individuals must continuously process motion-related information, such as approaching pedestrians or passing vehicles when crossing a street, and make safe behavioral decisions based on this information. In competitive sports, the demands on motion-information processing are even more pronounced (Scharfen & Memmert, 2019). In table tennis, for example, the ball travels at high speeds, lands unpredictably on multiple points, and exhibits varying degrees of spin. These characteristics place exceptional demands on players’ visual information processing, reaction speed, motor-control precision, and decision-making abilities.

Experience plays a crucial role in enhancing an individual’s capacity to process information. Long-term physical training promotes motor performance, optimizes the visuomotor cortical network, and improves cortical neural efficiency, consistent with the principles of brain plasticity (Zhang et al., 2025). Prolonged, sport-specific training induces adaptive structural and functional changes in the cerebral cortex, thereby enhancing athletes’ motor skills and cognitive abilities (Logan et al., 2022). A scoping review with meta-analysis on EEG research found that the favorable brain function in athletes can be summarized as neural efficiency, increased cortical asymmetry, greater cognitive flexibility, and precise timing of cortical activation (Fang et al., 2022). Therefore, continuous professional skill training can further reshape brain architecture and function (Zhang et al., 2021), strengthen neural network plasticity, and ultimately improve the processing of motor-related information. Research has shown that elite athletes possess extensive procedural and declarative knowledge, enabling them to extract critical cues from complex environments (McPherson, 2000). Differences in motor-information processing between athletes and non-athletes are also evident at the neural level. According to the neural efficiency hypothesis (NEH), experts exhibit more efficient cortical functioning during cognitive tasks (L. Li & Daniel, 2021). For instance, compared with amateurs and young elite, researchers has found that expert table tennis players are characterized by enhanced cortical activation in the motor and fronto-parietal cortex during motor imagery in response to table tennis videos (Wolf et al., 2014). In addition, elite expert players in table tennis have less activation of the fronto-parietal attention network (Wolf et al., 2014). Greater neural efficiency is typically reflected in reduced activation of task-relevant brain regions and enhanced inhibition of irrelevant neural activity (Qiu et al., 2019). In electrophysiological studies, larger P3 amplitudes indicate a greater allocation of cognitive resources to the task at hand (Key et al., 2005). The N2 component, which appears around 200–400 ms with a frontal-midline maximum, displays more negative amplitude in tasks involving perpetual conflict and response inhibition (Kousaie & Phillips, 2017). In other word, larger N2 amplitudes are usually associated with enhanced inhibition.

You et al. (2018) compared response inhibition between table tennis players and non-athletes under both conscious and unconscious conditions. Their findings showed that athletes exhibited larger P3 amplitudes in No-Go trials across both conditions, indicating stronger inhibitory control and higher neural efficiency. Another study reported that during tasks involving motion anticipation and visuospatial memory, high-speed stimuli elicited larger P3 amplitudes; however, the experienced group demonstrated shorter P3 latency, suggesting more rapid working-memory updating and more efficient decision making (Chueh et al., 2017). A study reported that ERP N2 amplitudes, or N2 or P3 latencies, the elite and expert groups exhibited notably faster reaction times and more pronounced P3 amplitudes than the intermediate group did during the cognitive task (Chang et al., 2024). Long-term training also strengthens the cerebral cortex’s capacity to regulate motor actions (Patel & Azzam, 2005). Research has further shown that table tennis players exhibit shorter N2 latency under all conditions, reflecting faster conflict-monitoring processes (You et al., 2018). Using a masking paradigm, another study examined athletes’ neural efficiency in processing spin-serve information at an unconscious level. The results revealed markedly reduced N2 amplitudes under incongruent conditions, indicating superior conflict monitoring and response-suppression abilities (Shi et al., 2024). Huang et al. (2024) investigated behavioral performance and neural activity in table tennis players during domain-specific (color-word Stroop) and spatially based (spatial Stroop) tasks. They found that athletes displayed lower N2 amplitudes, suggesting that they required fewer neural resources for conflict detection and resolution, evidence that long-term training optimizes the brain’s inhibitory-control network. In general, larger N2 amplitudes reflect heightened cognitive conflict and a greater need for response inhibition.

Taken together, previous research demonstrates that motor experience can enhance neural efficiency and improve the processing of motor-related information. However, studies examining how varying levels of experience influence motor-information processing from the perspective of neural activity remain limited (Chen et al., 2024). The majority of studies have only compared the behavioral and neural mechanisms between elite athletes and non-athletes within a specific sport (Moscatelli et al., 2016), or have merely examined the differences between athletes and non-athletes in processing general movement information (Song et al., 2024). Few studies examined the differences in how elite athletes, amateur athletes, and non-athletes processed sport-related information.

To address this gap, the present study recruited college students with different levels of table tennis experience, including table tennis specialists, athletes from other sports, and non-sports majors, to participate in an electroencephalogram (EEG) experiment. Participants freely viewed a series of images while their electroencephalographic activity was recorded. These images were taken from screenshots of table tennis matches. Competitive images were extracted from the athletes’ official competition period, and non-competitive images were captured from their pre-competition training sessions. Our objective was to investigate the differences in neural responses to competitive and non-competitive images among three distinct groups, in order to elucidate the role of motor experience in the processing of motor-related information. We hypothesize that motor experience would modulate the processing of motion information, reflected in differential N2 and P3 amplitudes across groups. Specifically, table tennis players and ordinary athletes may have extensive training in their specific sports, which results in a smaller amplitude of the P3 component compared to non-athletes. That is to say, they can effectively process sports-related information without requiring a lot of cognitive resources. For the passive viewing of competitive images, since the images are related to the information of table tennis in the competition scene, table tennis players may be more sensitive and show differences in the amplitude of the N2 component compared to non-athletes. The findings of this study are expected to provide empirical support for the NEH.

## 2. Materials and Methods

### 2.1. Participants

A two-factor mixed experimental design of 3 (sports experience: table tennis players, ordinary athletes, non-athletes) × 2 (image type: competitive vs. non-competitive) was employed. The required sample size was estimated using G*Power 3.1.9.2 (α = 0.05, 1 − β = 0.90, effect size f = 0.25), indicating a minimum of 54 participants. Ultimately, 60 college students were recruited, with 20 individuals assigned to each group. Table tennis players were required to engage in at least 10 h of professional training per week. This group included 15 male students (*M* = 21.47, *SD* = 1.52) and 5 female students (*M* = 21.60, *SD* = 1.28). Ordinary athletes, who had no table tennis experience, also trained for no less than 10 h per week. This group comprised 14 males (*M* = 21.33, *SD* = 1.50) and 6 females (*M* = 21.83, *SD* = 0.41). The non-athlete group consisted of 20 students without any formal sports training experience, including 5 males (*M* = 21.75, *SD* = 0.96) and 15 females (*M* = 21.40, *SD* = 2.38). All participants were in good physical health, reported no history of psychiatric or neurological disorders, and were right-handed. Before the experiment, written informed consent was obtained from all participants, and they received monetary compensation upon completion of the study. The experimental protocol was approved by the university’s ethics committee (Approval No.: LL2024193).

### 2.2. Stimuli and Procedure

The experimental materials consisted of 150 images extracted from various table tennis matches. These images were categorized into two conditions: competitive, in which the athlete was depicted in an active competitive state, and non-competitive, in which the athlete was shown in a practice or training context. To validate the categorization, 25 table tennis players who did not participate in the main experiment rated the images. Based on their evaluations, 30 images with low discriminability between competitive and non-competitive states were removed. The final stimulus set comprised 120 images, including 60 competitive and 60 non-competitive pictures. The competitive-state ratings (1–5 scale) showed a significant difference between the two categories, *t* = 3.68, *p* < 0.05.

**Figure 1:**
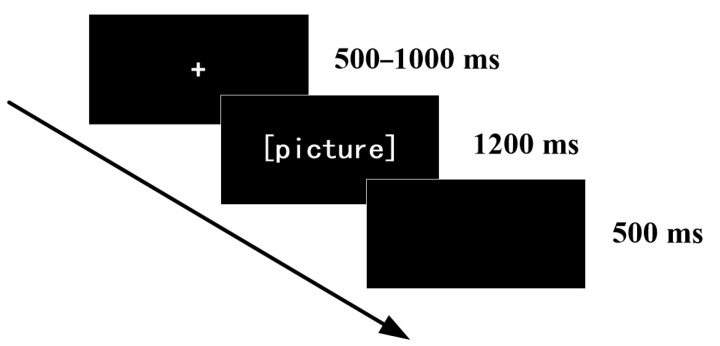
The process of a trial.

The experiment consisted of two phases. Upon arrival at the laboratory, participants were given a brief rest period, after which the experimenter provided standardized instructions. They then completed eight practice trials to familiarize themselves with the stimulus materials and task procedure. Following the practice phase, the formal experiment began. Each trial started with the presentation of a fixation cross (“+”) for a jittered duration of 500–1000 ms, followed by a table tennis-related image displayed for 1200 ms. Participants were instructed to view each image attentively. After the image disappeared, a blank screen was shown for 500 ms, marking the end of the trial (see Figure 1). To ensure that the participants would pay close attention to the images, randomly in 20% of the trials, a screen with a question mark appeared after the image was shown, and the participants were required to respond by pressing a “F/J” key to indicate whether the image depicted a competitive state or a non-competitive state. The formal experiment comprised 120 trials in total. The whole experiment was divided into 4 blocks, each block consisting of 30 trials. The participants taken a short break after each block. After adjusting their own state, they would proceed to the next block of the experiment.

### 2.3. EEG Recording

EEG data were recorded using a 64-channel Brain Products system. Electrode FPz served as the ground, and FCz was used as the online reference. Vertical electrooculogram activity was recorded using an electrode placed below the right eye to monitor blinks and vertical eye movements. Electrode impedances were maintained below 5 kΩ throughout the recording, and EEG signals were digitized at a sampling rate of 1000 Hz.

Offline preprocessing was performed using BrainVision Analyzer 2.0 (Brain Products GmbH, Gilching, Germany). The data were re-referenced to the average of the left and right mastoids and filtered using a 0.1–30 Hz bandpass filter (24 dB/octave). Ocular artifacts were corrected with independent component analysis. The corrected EEG was segmented into epochs from −200 ms to 800 ms relative to stimulus onset, with the −200 to 0 ms interval used for baseline correction. Epochs containing artifacts exceeding ±100 μV in peak-to-peak amplitude were rejected. After artifact removal, the mean numbers of usable trials were as follows: for table tennis players, 50.88 (*SD* = 6.98) in the competitive condition and 51.41 (*SD* = 7.11) in the non-competitive condition; for ordinary athletes, 52.68 (*SD* = 3.87) in the competitive condition and 52.12 (*SD* = 4.40) in the non-competitive condition; and for non-athletes, 52.06 (*SD* = 5.72) in the competitive condition and 52.11 (*SD* = 6.00) in the non-competitive condition.

### 2.4. Data Analysis

**Figure 2:**
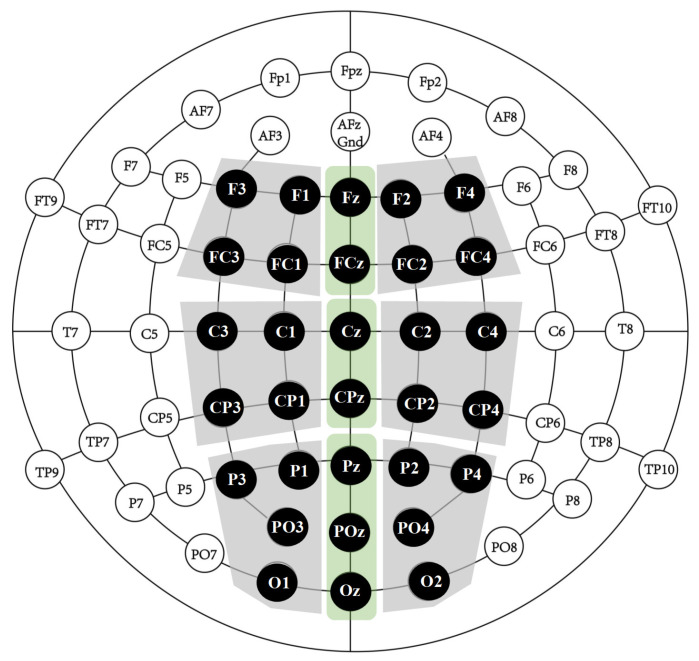
Topographical clusters and electrode positions. The green and gray areas demonstrates the midline cluster and the other six clusters.

Based on previous studies (Chueh et al., 2017; Shi et al., 2024), the N2 component was analyzed within the 220–270 ms time window at frontal and central electrode sites, while the P3 component was analyzed within the 300–460 ms window at central and parietal sites. The regions of interest are illustrated in Figure 2 and included the following electrode clusters: left-anterior (F1, F3, FC1, FC3), middle-anterior (Fz, FCz), right-anterior (F2, F4, FC2, FC4), left-central (C1, C3, CP1, CP3), middle-central (Cz, CPz), right-central (C2, C4, CP2, CP4), left-posterior (P1, P3, PO3, O1), middle-posterior (Pz, POz, Oz), and right-posterior (P2, P4, PO4, O2).

The N2 amplitudes were analyzed using two separate 3 × 2 × 3 mixed-design ANOVAs with the factors condition (competitive vs. non-competitive) and hemisphere (left, middle, right) as within-subject variables, and group (table tennis players, ordinary athletes, non-athletes) as the between-subject factor. These analyses were conducted separately for frontal and central sites. Similarly, two 3 × 2 × 3 additional mixed-design ANOVAs were conducted on P3 amplitudes for central and posterior sites, respectively, with the same within- and between-subject factors.

For all ANOVAs involving more than one degree of freedom, the Greenhouse-Geisser correction was applied to adjust for violations of sphericity. Post hoc pairwise comparisons were corrected using the Bonferroni method to control for multiple comparisons. Statistical significance was set at an alpha level of 0.05 (two-tailed).

## 3. Results

**Figure 3:**
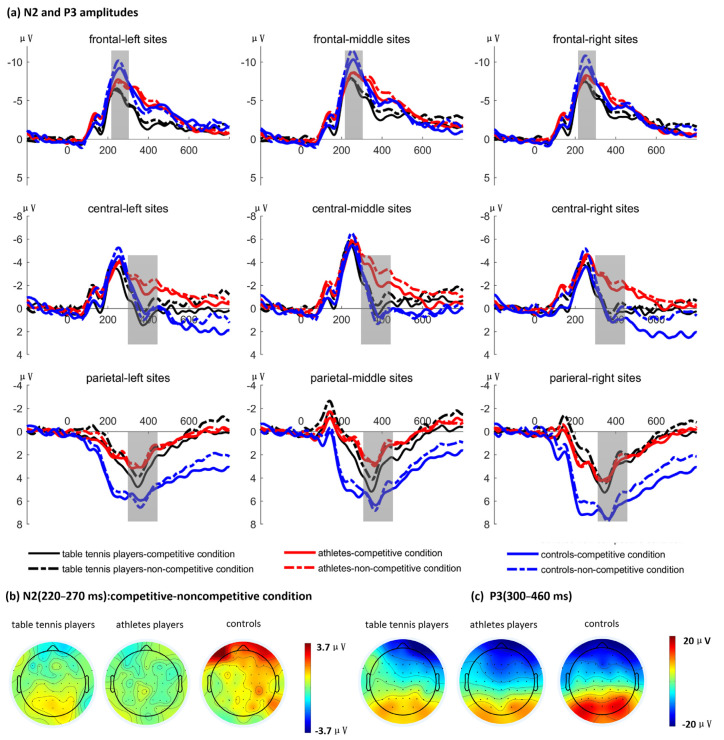
The waveforms of ERPs in different sites. (**a**) Grand averaged waveforms evoked by stimuli. (**b**) Topographies showing the average amplitude differences between competitive and non-competitive condition for N2 component. (**c**) Topographies of the average amplitude differences between conditions for P3 component.

Table 1 showed the average amplitudes of ERPs and Figure 3 showed the grand average waveforms of ERPs in different sites.

Prior to conducting the repeated-measures ANOVA, the assumption of normality was assessed. we examined the skewness and kurtosis statistics. The absolute values of their corresponding z-scores (calculated by dividing the statistic by its standard error) were all below the critical value of 1.96 for three groups at each condition, indicating no significant departure from normality. The results found that the data within each group at each condition (competitive and non-competitive separately) were normally distributed for N2 amplitudes in frontal sites (all |Z| < 1.82), as well as for P3 amplitude in central (|Z|s < 1.75) and parietal sites (|Z|s < 1.95). Moreover, the data within each group at non-competitive condition were normally distributed for N2 amplitudes in central sites (non-competitive:|Z|s < 1.61). For the competitive condition, although the skewness z-score for the athlete group slightly exceeded the threshold (Z = 2.37), visual inspection of the Q-Q plots indicated an acceptable alignment with the normal distribution. The data of other two groups were normally distributed (|Z|s < 1.89).

### 3.1. N2 Amplitude

As shown in Figure 3, a significant interaction between group and condition was observed at the frontal sites, *F* (2, 57) = 3.26, *p* = 0.046, *η_p_*^2^ = 0.10. Post hoc analyses revealed that, for the non-athletes group, the competitive condition elicited significantly smaller N2 amplitudes compared to the non-competitive condition (Δ = 1.17, 95% CI [0.36, 1.97], *p* = 0.005). In contrast, no significant differences between conditions were found for either the table tennis players or the ordinary athlete groups (*p*s > 0.78). The main effect of group was not significant, *F* (2, 57) = 1.42, *p* = 0.25, and the main effect of condition did not reach significance, *F* (21, 57) = 2.11, *p* = 0.15. All other interactions were not significant (*p*s > 0.21).

At the central sites, the main effect of condition was significant, *F* (1, 57) = 6.03, *p* = 0.02, *η_p_*^2^ = 0.10, indicating that the competitive condition elicited smaller N2 amplitudes compared to the non-competitive condition. The main effect of group was not significant, *F* (2, 57) = 0.04, *p* = 0.97. No other interactions reached significance (*p*s > 0.07).

### 3.2. P3 Amplitude

At the central sites, there was a marginally significant main effect of condition, *F* (1, 57) = 3.93, *p* = 0.052, *η_p_*^2^ = 0.06, with the competitive condition eliciting larger P3 amplitudes compared to the non-competitive condition. But the main effect of group was not significant, *F* (2, 57) = 1.39, *p* = 0.26. No other significant main effects or interactions were observed (*p*s > 0.12).

At the parietal sites, a significant main effect of condition was found, *F* (1, 57) = 4.73, *p* = 0.034, *η_p_*^2^ = 0.08, indicating that the competitive condition elicited larger P3 amplitudes than the non-competitive condition. Additionally, there was a significant main effect of group, *F* (2, 57) = 3.66, *p* = 0.03, *η_p_*^2^ = 0.11. Post hoc comparisons revealed that controls elicited larger P3 amplitudes than table tennis players (Δ = 2.77, 95% CI [0.04, 5.50], *p* = 0.047) and larger amplitudes than ordinary athletes (Δ = 3.50, 95% CI [0.77, 6.23], *p* = 0.01). No significant difference was observed between table tennis players and athletes (*p* = 0.60). Other main effects and interactions were not significant (*p*s > 0.22).

## 4. Discussion

### 4.1. Analysis of the Meaning of the Research Results

This study primarily investigated the influence of professional sports experience on the neural processing of sport-related information using ERPs. Participants were categorized into three groups based on their level of sports experience: table tennis players, ordinary athletes, and non-athletes. The experimental stimuli consisted of screenshots extracted from table tennis matches, depicting athletes and ball movements in either competitive or practice (non-competitive) states.

Participants passively viewed the images while their EEG responses were recorded. The results revealed that competitive conditions elicited larger P3 amplitudes in parietal regions and smaller N2 amplitudes in central regions compared to non-competitive conditions. Notably, non-athletes produced larger P3 amplitudes in the parietal region than both table tennis players and ordinary athletes. Furthermore, non-athletes exhibited smaller frontal N2 amplitudes in response to competitive conditions relative to non-competitive conditions, a difference not observed among athletes.

These findings suggest that professional sports experience modulates the neural mechanisms underlying the processing of sport-related information. Specifically, both table tennis players and ordinary athletes demonstrated reduced P3 amplitudes in parietal regions compared to non-athletes, indicating more efficient cognitive resource allocation during the processing of competitive scenarios. an ERP research also found that sanda athletes had significantly larger amplitudes for the N200 and P300 components in incongruent trials compared to congruenttrials, indicating that Sanda athletes enhanced cognitive control abilities (Y. Li et al., 2025). The P3 component is widely associated with attentional resource allocation; thus, increased P3 amplitude among non-athletes likely reflects greater cognitive effort required to process motor information, consistent with lower neural efficiency (Key et al., 2005). A study on the decision-making of athletes showed that elite athletes had smaller P3 amplitudes in their decision-making compared to amateur athletes (Xiong & Song, 2024). The researchers also believed that the P3 component was associated with the allocation of cognitive resources. The lower P3 peak amplitudes in the expert group suggested that experts required less neural activation to process and respond to stimuli, indicating a more streamlined and efficient cognitive processing mechanism.

In this study, non-athletes exhibited smaller N2 amplitudes in the central region under competitive conditions compared to non-competitive conditions. In contrast, no significant difference in N2 amplitude was observed between competitive and non-competitive conditions for either table tennis players or ordinary athletes. This pattern suggests that motor experience plays a similar role in processing various types of motion information and may produce a transfer effect across contexts. The N2 component, typically observed in frontal and central brain regions, is generally associated with conflict monitoring and response inhibition (Nakamoto & Mori, 2012). Larger N2 amplitudes often indicate increased cognitive conflict and a greater need for inhibitory control. The differential N2 amplitude responses between competitive and non-competitive conditions seen in non-athletes may reflect their limited sports experience, resulting in less efficient conflict monitoring and response inhibition during sports information processing. Conversely, the absence of N2 amplitude differences in athletes suggests a higher neural efficiency, allowing them to process competitive and non-competitive sports information without requiring additional cognitive resources. In competitive scenarios, non-athletes may need to exert extra effort to suppress attention to irrelevant stimuli, such as crowd noise or distractions, which could manifest as reduced N2 amplitude. Athletes, through extensive training, develop stable inhibitory control abilities (Nakamoto & Mori, 2012). For example, previous research using conflict tasks (e.g., Go/No-go) has shown that athletes exhibit smaller frontal N2 amplitudes, reflecting enhanced suppression of irrelevant information. The “experience template” developed by table tennis players likely reduces conflict perception, as evidenced by stable N2 amplitudes across conditions. This inhibitory control advantage may be linked to the unique demands of table tennis, which requires rapid adaptation to constantly changing competitive scenarios (Shi et al., 2024).

Notably, this study found that table tennis players and ordinary athletes exhibited similar EEG responses when processing competitive and non-competitive stimuli in sports scenarios. This suggests that long-term training enhances athletes’ cognitive processing abilities related to sports information, including attention, visual search, and memory. In addition, no differences were observed in the behavioral outcomes and neural correlates of cognitive control abilities between open-skill sports athletes and closed-skill sports athletes, reflecting the neural similarity among athletes engaged in different types of training (Veliks et al., 2024). The similarity in ERP patterns between the two athlete groups may reflect a transfer or migration effect, where cognitive skills developed through sports training generalize across different domains. Such enhanced cognitive abilities, like attentional allocation and visual search, are believed to possess domain-general qualities (Logan et al., 2022). Moreover, common elements across sports, such as skill acquisition, tactical strategies, and motor techniques, may facilitate the transfer of these cognitive enhancements to various competitive fields.

### 4.2. Limitations of the Study and Future Research Directions

This study has several limitations. First, the experimental materials consisted solely of static images depicting competition scenes, which differ substantially from real-life dynamic situations. Static images may not fully engage specific perceptual processes, such as judging the rotation of a table tennis ball. Future research should employ dynamic stimuli, such as videos, to better capture the electrophysiological responses involved in processing motion information under competitive and non-competitive conditions (Chen et al., 2024).

Second, the experimental task was relatively simple, involving only passive viewing. Although the study demonstrated that sports experience influences the neural processing of sports information, it did not reveal distinct differences between table tennis players and ordinary athletes. Moreover, the decrease in the amplitude of the N2 wave is difficult to be solely attributed to an increase in neural activity efficiency or insufficient inhibitory capacity. Future studies should incorporate more demanding tasks, particularly those requiring active judgment of sport-specific information related to table tennis, to further investigate the specialized processing characteristics of expert athletes within their domain. The significance of changes in N2 amplitude can be verified through behavioral data. A decrease in N2 amplitude when behavioral response improves can be presumed to indicate an increase in neural activity efficiency, while a decrease in N2 amplitude when behavioral response decreases can be presumed to suggest insufficient inhibitory control.

Thirdly, there is a gender composition bias among the three groups of subjects in this study. There are more male participants in the two athletes groups and more female participants in the non-athlete group. The results cannot rule out the role of gender factors, or there is a risk in interpreting the results as the role of motor experience. Future research will need to control the gender ratio among different groups.

## 5. Conclusions

To investigate the influence of sports experience on the processing of sports information, participants were divided into three groups based on their level of athletic experience. The stimuli included images depicting both competitive and non-competitive table tennis scenarios. The results demonstrate that long-term physical training enhances the brain’s ability to process motor information and improves cognitive efficiency. Specifically, athletes allocate cognitive resources more effectively and exhibit stronger conflict monitoring and response inhibition, leading to more efficient processing of sports-related information. Additionally, these cognitive benefits appear to transfer across different sports experiences.

## Figures and Tables

**Table 1:** Means (*SD*s) of ERPs in different sites.

Component	Site	Hemisphere	Competitive			Non-Competitive		
			Players	Athletes	Controls	Players	Athletes	Controls
N2	frontal	left	−6.43(4.78)	−7.39(5.46)	−8.80(5.40)	−6.21(4.39)	−7.15(5.14)	−7.69(5.23)
		middle	−7.68(4.94)	−8.17(5.74)	−9.79(6.84)	−7.68(4.59)	−8.25(5.33)	−10.96(6.35)
		right	−7.26(4.69)	−7.80(5.41)	−8.93(5.47)	−7.34(4.75)	−7.61(5.45)	−10.35(5.29)
	central	left	−3.29(4.79)	−3.73(4.60)	−4.28(8.07)	−3.90(4.39)	−3.83(4.00)	−4.97(8.17)
		middle	−5.35(5.01)	−5.37(5.68)	−5.16(7.05)	−5.71(4.79)	−5.66(5.30)	−6.13(6.93)
		right	−3.65(4.95)	−4.24(3.88)	−3.36(7.01)	−4.17(4.66)	−4.10(3.91)	−4.83(6.05)
P3	central	left	0.50(5.67)	−1.86(3.93)	−0.47(6.58)	−0.58(5.37)	−2.47(4.06)	−0.10(7.45)
		middle	−0.51(5.85)	−2.71(4.69)	−0.42(5.88)	−1.24(5.48)	−3.63(5.24)	−0.03(6.09)
		right	0.22(5.57)	−2.12(3.10)	0.56(5.96)	−0.40(5.01)	−2.51(3.92)	−0.05(5.26)
	parietal	left	3.93(5.01)	2.76(3.02)	6.18(6.27)	2.98(4.72)	2.39(2.94)	6.27(6.32)
		middle	3.62(5.42)	2.08(2.61)	5.57(5.25)	2.60(5.03)	1.87(3.03)	5.47(4.23)
		right	3.83(4.67)	3.38(6.79)	6.79(5.61)	3.09(4.24)	3.23(3.28)	6.42(4.85)

Note: Data are expressed as mean (*SD*). *SD*: standard deviation.

## Data Availability

Dataset available on request from the authors.

## Abbreviations

The following abbreviations are used in this manuscript:

EEG	Electroencephalogram
NEH	Neural efficiency hypothesis
